# The Analysis of Inorganic Bodies

**Published:** 1833-10-01

**Authors:** 


THE ANALYSIS OF INORGANIC BODIES. By *Professor Berzelius.* Translated from the French, by *G. O. Rees.* London, 12mo, pp. 164.

EVERY work coming from the hands of so great a master of his art as Berzelius carries along with it its own recommendation. There is no need of any encomiums to introduce it to public favour; the name of its author is sufficient. The distinguished Swede is admitted by every one to be "facilè princeps" in the field of chemical analysis, to which he has given almost the exactness of mathematical research. The little volume now before us forms part of the 12th volume of the author's elementary chemistry; but as it forms a complete and very admirable treatise on that branch of the science which is devoted to the analysis of inorganic bodies, the translator has done well to detach it from the great work, for the benefit of those who engage in the practical details of this delightful study. To all such, it is an indispensable companion and instructor, pointing out what is to be done, and the manner of doing it. As well might a botanist go into the fields to collect and ascertain plants without a Flora in his pocket, as a chemical student enter upon the labours of the laboratory without a manual to direct him; and of all the manuals hitherto published, the one before us is alike the cheapest and the best.

In the present day, when the attention of medical men is once more specially directed to the composition of the animal fluids, and when the foundations of a rational and restricted humorism are probably again laid, or are in the act of being so; and moreover, now that the standard of professional qualifications is daily becoming more and more scientific, as appears from the existing rivalry of the different schools and universities throughout the kingdom, it is highly important, if not absolutely necessary, that a competent knowledge of manual chemistry be acquired by all. We are glad to observe that this study is becoming more popular, and that numerous classes have recently been established for its institution. If the knowledge of the science be considered, as it most properly is by all, a necessary branch of medical education, surely a practical acquaintance with the facts on which the science is built, or, in other words, with the art of chemistry, is equally or still more so.

Do we not recognise the truth of this position in the cases of anatomy and of materia medica? Do we not require that the student take the scalpel into his hand, and diligently and patiently examine for himself every part, that he may know them by sight and by feeling, as well as that he may describe them with accuracy and minuteness? And again, are we satisfied with his skilful answers about the names, localities, uses, compositions, and so forth, of the different drugs in the Pharmacopœia? No, we expect and insist that he should have handled each and all of them, that he can recognize them at once, and be acquainted with the manipulations of preparing and of exhibiting them. Surely, then, it is of equal, or rather of still higher importance, that he be well informed in the details of practical chemistry. How, we ask, can a medical jurist dispense with it? and none of us can know when we may be called upon to act as such. Is not the army and naval surgeon frequently desired to investigate and report upon cases of poisoning? and how can they do so, unless they know the tools that they must work with, and the right method of using them?

But even in ordinary and every-day practice, a scientific physician requires his test-tubes and his box of re-agents. In diseases of the urinary organs, a knowledge of the chemical changes in the water is of the first consequence; in dropsy it is equally so; and full well we all remember its interesting applicability to the etiology of cholera.

As a sample of this deserving volume, we select the catalogue of rules to be followed in the examination of mineral waters by re-agents;—premising, that they are equally illustrative of the analysis of urine, or of other animal secretions. We are directed first to observe the colour and transparency of the fluid; its smell, especially after it has been well shaken; and its taste, which may be saline, bitter, or like that of ink.

We should then take thirteen glasses, or test tubes, "ten are filled with the fresh water for examination, and three with prepared water that has been boiled at least for half an hour, and that has been filtered after cooling. The following re-agents are next dropped into the glasses:—

*First.*—Tincture of turnsole (prepared with cold alcohol and cakes of turnsole: alcohol preserves the tincture unaltered for a length of time.) When the water into which a few drops of this tincture have been thrown assumes a red shade, the presence of a free acid must be concluded: if, on the addition of a greater quantity of tincture, the reddish liquor assumes a bluish tint, the quantity of acid is small; and if the reddened water becomes blue again after from twelve to twenty-four hours, and the tincture of turnsole added to one of the glasses containing the boiled mineral water does not become red, the acid contained in the water was the carbonic: a dark permanent red colour denotes the presence of a metallic salt.

*Second.*—Lime water saturates free carbonic acid, so as to occasion a precipitate of neutral carbonate of lime: the earths and metallic oxides which had been dissolved in the carbonic acid precipitate at the same time. If the water contain free carbonic acid, the precipitate is redissolved by the addition of a sufficient quantity of this water. In order to make the experiment well, it is commenced by allowing a few drops of lime-water to fall into the water: the troubling which is at first occasioned afterwards disappears. If the water do not contain free carbonic acid, but only bicarbonates, the troubling caused by the lime water does not disappear, whatever quantity of the mineral water may be added. The majority of Swedish waters that I have examined, containing carbonic acid, act in this manner.

*Third.*—The tincture of Brazil wood passes from a yellowish brown to a beautiful red when the mineral water contains an alkali or an earthy carbonate.

*Fourth.*—The chloride of barium precipitates a sulphate of barytes. An alkaline water ought to be mixed with acid, before adding the barytic solution to it, so as to prevent the re-action of the alkali on the barytic salt: few Swedish waters contain sulphates in considerable quantity.

*Fifth.*—Nitrate of silver indicates the presence of the chlorides, which precipitate it in the form of a white and thick cloud. If in the first instance the precipitate is black or brown, the presence of sulphuretted hydrogen may be concluded. Sometimes after a short interval the supernatant liquor assumes a wine-red colour, still preserving its transparency. This effect, according to the experiments of M. Hermbstaedt, is owing to the presence of an acid and volatile body, which he supposes to be the sulphuretted or phosphuretted hydrogen: he has found it both in the water of the Baltic Sea, and in that obtained from it by distillation.

*Sixth.*—Oxalate of ammonia and the acid oxalate of potassa precipitate an oxalate of lime which deposits slowly.

*Seventh.*—The subphosphate of ammonia added to the filtered liquor shows the presence of magnesia.

*Eighth.*—Caustic potassa precipitates the earths and metallic oxides from their solution in acids. A white precipitate, which after some time becomes yellow, indicates the presence of iron, or else of a certain quantity of extractive matter that colours the precipitate.

*Ninth.*—Bicarbonate of potassa precipitates the earthy and metallic oxides, when dissolved in any other acid than the carbonic.

*Tenth.*—Ferro-cyanuret of potassium gives a green colour to alkaline ferruginous waters, and a greenish-blue precipitate deposits after some hours. If the water be not alkaline, or when the alkali has previously been saturated with an acid, the precipitate immediately assumes a blue colour. In the previously boiled water the ferro-cyanuret of potassium does not react when the iron has been held in solution by the carbonic acid.

The red ferro-cyanuret is still more sensible than the yellow one, because the springs contain iron in the state of protoxide, with which this last re-agent immediately forms a blue precipitate.

*Eleventh.*—The neutral deutochloride of gold produces a troubling in ferruginous waters, according to M. Ficinus; causing a precipitate of metallic gold, which is visible even in waters on which the prussiate of potassa and gall-nut produce no effect; but it is necessary that the free acid should be first saturated by carbonate of soda.

*Twelfth.*—Gallic acid, or, in lieu of this, the alcoholic infusion of nut-galls, does not at first produce any change in a ferruginous water just drawn, but after some time the water becomes gradually coloured. A clear purplish colour, which does not increase after some hours, indicates an exceedingly small quantity of iron. Our ferruginous waters generally give a very dark purple colour; and those which contain more iron assume a blackish-brown colour. Waters that contain much alkali give a dirty colour, between green and dark brown. Waters containing so little iron that its presence cannot be demonstrated by tincture of galls alone re-act sensibly, according to Mr. Philips, when a little lime water, or, what is better still, a solution of carbonate of lime in carbonated water, is added to them.

When gallic acid does not produce a purple colour in the boiled water, the protoxide of iron has been held in solution by the carbonic acid; and if the boiled water assumes, after some hours, a sea-green colour with the gallic acid, it contains some alkali.

This re-action is so delicate, that it serves to demonstrate the smallest quantities of alkali; but for that purpose it is necessary that the water should have been boiled some length of time, for otherwise the alkaline re-action may be owing to the presence of carbonate of magnesia.

A crowd of other re-agents exist, but their employment has never taught me any thing new; and those that I have enumerated have always sufficed." 85.

We are tempted to give another extract, which will no doubt be acceptable and useful to all, and more especially to such of our readers as are abroad, or who have not in their libraries all the more recent works on chemistry. The topic treated of is the detection of arsenic.

"In cases where the poison exists in the stomach in the fluid state, Valentine Rose recommends that the matter be boiled in water, containing a few grains of caustic potassa, and then filtered; the filtered solution is again boiled, and whilst in this state nitric acid is added, so long as any precipitate appears, and until the liquor becomes clear and yellow; the boiling solution is now filtered, and nearly saturated with carbonate of potassa, care being taken to drive away all carbonic acid. Lime water is then added, which combines with the excess of nitric acid, and precipitates with the arsenious acid, as arsenite of lime, but in a very impure state; this precipitate is washed on a filter and dried, and may be reduced by boric acid and charcoal, in the tube before-mentioned.

M. Berzelius recommends that the liquor be boiled in an alkaline solution, which is to be afterwards saturated with hydrochloric acid, and filtered; this liquor may now be tested with sulphuretted hydrogen, when, if arsenic be present, a yellow precipitate of sulphuret is produced, which is to be thrown upon a small quantity of fused nitre at the bottom of a tube; an arseniate of potassa is formed, which is dissolved in a little distilled water, and decomposed by a solution of lime, which precipitates arseniate of lime; it may be well to mention, that boiling much facilitates the formation of this precipitate. The test of reduction practised by M. Berzelius is performed with charcoal only, for though boric acid, when added, causes the sublimation to be produced at a much lower temperature, yet it swells so much by heat, that it should be avoided as much as possible in delicate operations. In using hydrochloric and sulphuric acids, care should be taken that they contain no arsenic, which is sometimes the case when they are prepared with arsenical pyrites; this source of fallacy is easily avoided by testing the acid re-agents with a stream of sulphuretted hydrogen.

The processes of Berzelius and Rose are in many points objectionable, and do not equal that proposed by Dr. Christison, either in simplicity or accuracy; thus, the arseniate of lime is partly lost during the washings, being far from insoluble in water; and the arseniate, when heated with charcoal, even by the full red heat of a blow-pipe, only yields one-third of its arsenic in the metallic state. Dr. Christison's process consists in boiling the mass in distilled water for half an hour, (which he has satisfactorily proved is capable of dissolving the arsenic present, provided the matter examined be previously cut into shreds): it is then filtered and acidulated with acetic acid, which precipitates the casein, &c.; the liquor is again filtered and treated with sulphuretted hydrogen; the sulphuret is collected on a filter, and reduced with black flux, in a tube similar to that employed by Berzelius and Rose. Before treating the liquor with sulphuretted hydrogen, it is well to see if the ammoniacal nitrate of silver give its characteristic yellow colour with the solution, which effect, if produced, will indicate that it is ready for the passage of the gas, but if it do not act satisfactorily, a necessity will be indicated for the further purification of the liquor, which may be effected by simply evaporating it to dryness, and boiling the residue with distilled water, when a liquor will be procured, which may be tested with the sulphuretted hydrogen. As regards the quantity of sulphuret capable of giving satisfactory evidence of the presence of arsenic, I have produced a perfectly characteristic metallic crust from a portion of sulphuret not larger than half a grain of mustard seed; and this when freshly precipitated and dried, and therefore in a loose and uncondensed form." 117.

Mr. Rees has translated the work from the French edition with much neatness and accuracy. There is only one defect we have observed in the perusal of it, and that is, the want of an index. In a book which is intended to be a manual, and therefore one of very frequent reference, every facility should be afforded the student to find out, in a moment, the passage which he is in search of. In a second edition, Mr. Rees will no doubt attend to this suggestion.

